# Life review on psycho‐spiritual outcomes among older adults with life‐threatening illness: A systematic review

**DOI:** 10.1093/geroni/igaa057.3316

**Published:** 2020-12-16

**Authors:** Mandong Liu, Ying Wang, Iris Chi

**Affiliations:** 1 University of Southern California, Los Angeles, California, United States; 2 Lanzhou University, Lanzhou, Gansu, China

## Abstract

Life review has been widely conducted to help older adults enhance well-being and cope with burdens. Spirituality is an important part of an older adult’s overall well-being, especially for those with life-threatening illnesses. However, there is no review study examining the effectiveness of life review interventions on psycho-spiritual outcomes among this population. The aim of the study was to examine the effectiveness of life review on psycho-spiritual well-being among older adults with life-threatening illnesses. A systematic review with meta-analysis consistent with the recommendations of the Cochrane Collaboration was conducted. Database searches included PubMed, PsycINFO, the Cochrane Library, the Campbell Library, EBSCO, CNKI, and the Airiti Library up to March 2020. Grey literature and reference lists from relevant articles were also searched and reviewed. 34 studies were included in the systematic review and 33 were included in the meta-analysis for outcomes of anxiety, depression, and quality of life. Other psycho-spiritual outcome measures that were not included in the meta-analysis due to the small number of studies included mood, apathy, life satisfaction, meaning in life, spiritual well-being, and some multi-dimensional instruments. The studies greatly varied in program design, content, format, length, interventionist, and more. The meta-analyses demonstrated standardized mean differences in favor of life review compared with the control with small to moderate effect sizes. The reviewers call for including more psycho-spiritual well-being measures among interventions for older adults with life-limiting illnesses, as well as studies with adequate sample size and rigorous designs in future research.

